# 

**Published:** 1881-02

**Authors:** 


CONTENTS.

| | Page. | | Page. |
|---|---|---|---|
| Ventilation | 39 | Superstitions about Cats | 60 |
| Principles of Medical Practice | 42 | How to Get a Wife in India | 61 |
| Dieting | 45 | Manifest Destiny of the English Race | 62 |
| Malaria (poetry) | 46 | Origin of the Morgue | 63 |
| Athletic Exercises | 47 | The Hard Road to Greatness | 64 |
| Clothing and Diet of Children | 49 | Men and their Children | 65 |
| Origin of the Merino Sheep | 51 | New and Stale Bread | 66 |
| The Foster-Brothers | 52 | Does Eucalyptus Prevent Fever | 67 |
| The Lumber Supply of Washington Territory | 59 | Waste of Horse Life | 71 |
| Robert Burns (poetry) | 59 | The Feet | 74 |

